# Editorial: Microbial involvement in biogeochemical cycling and contaminant transformations at land-water ecotones, volume II

**DOI:** 10.3389/fmicb.2026.1780529

**Published:** 2026-02-02

**Authors:** Yizhi Sheng, Xiangfeng Zeng, Linduo Zhao, Yongbin Li

**Affiliations:** 1 Center for Geomicrobiology and Biogeochemistry Research, State Key Laboratory of Geomicrobiology and Environmental Changes, China University of Geosciences, Beijing, China; 2 Key Laboratory of Pollution Ecology and Environmental Engineering, Institute of Applied Ecology, Chinese Academy of Sciences, Shenyang, Liaoning, China; 3 Prairie Research Institute-Illinois Sustainable Technology Centre, University of Illinois at Urbana Champaign, Champaign, IL, United States; 4 Key Laboratory of Industrial Ecology and Environmental Engineering (Ministry of Education), School of Environmental Science and Technology, Dalian University of Technology, Dalian, China

**Keywords:** bioremediation, land-water ecotones, microbial biogeochemistry, microbial community, mineral-microbe interaction

Land-water ecotones, including riparian zones, river-lake interfaces, estuaries, wetlands, and connected groundwater-surface water and other systems, represent some of the most dynamic and biogeochemically active environments on Earth. These transitional zones are characterized by steep physicochemical gradients, strong hydrological variability, and intense human disturbance, making them critical hotspots for evolution of life, elemental cycling and contaminant transformation. Microorganisms play a central yet often underestimated role in mediating carbon (C), nitrogen (N), phosphorus (P), sulfur (S), iron (Fe), and trace metal cycling within these ecotones, thereby regulating ecosystem evolution, functioning, water quality, and environmental resilience.

This Research Topic was initiated to synthesize recent advances in understanding how microbial communities respond to environmental gradients, hydrological connectivity, and anthropogenic pressures across diverse environment and geological time, and how these responses feedback on evolution of life, biogeochemical processes and contaminant fate (Sheng et al., 2024). The contributions published in this collection span diverse environments and methodological approaches, collectively highlighting the integrative roles of microbes at the intersection of microbiology, ecology, hydrology and geochemistry.

A recurring theme across several studies is the strong spatial structuring of microbial communities and functions along vertical and horizontal gradients in ecotonal environments. Depth-resolved analyses in semi-arid riparian soils revealed pronounced declines in microbial diversity and key functional genes involved in carbon fixation, denitrification, and phosphorus metabolism with increasing soil depth, underscoring the importance of surface soils as biogeochemical “active layers” (Liu, Wang et al.). These depth-dependent patterns were tightly coupled with soil porosity, mineral types, and nutrient availability, emphasizing the joint control of physical structure, mineral surface-mediated reactions, and resource distribution on microbial metabolic potential (Qiao et al., 2025; Sheng et al., 2022). Similarly, studies in river-lake connected systems and plain river networks demonstrated that spatial heterogeneity in nitrogen sources and pollution types exerts strong selection pressures on microbial community composition and ecological network complexity (You et al.).

Hydrological connectivity emerged as another critical regulator of microbial processes in land-water ecotones. The spatiotemporal distribution of microbial communities and functional potential displayed strong responses to the seasonal hydrogeochemical variations (Qiao et al., 2023; Chen et al., 2024). Seasonal shifts in river-lake connectivity not only reshaped nitrogen pollution sources but also indirectly altered microbial diversity and community structure through changes in nutrient loading and redox conditions. Field-based evidence further showed that hydrological disturbance, such as drying-rewetting cycles in streams, can profoundly reorganize microbial communities and their interaction networks (Chen et al.). While prolonged drying reduced bacterial richness and altered functional profiles, microbial communities displayed remarkable resilience upon flow resumption, supported by functional redundancy and the presence of functionally analogous taxa (Lin et al.). These findings highlight that ecosystem stability in fluctuating hydrological regimes is maintained not by taxonomic constancy, but by the persistence of key microbial functions embedded within complex ecological networks.

Another major focus of this Research Topic is the role of microorganisms in contaminant transformation and risk modulation in ecotonal sediments and aquifers. Several studies addressed organic and inorganic pollutants commonly associated with industrial, agricultural, and urban activities (Sheng et al., 2018; Jiang et al., 2023). In estuarine and coastal sediments, high levels of arsenic and polycyclic aromatic hydrocarbons (PAHs) were shown to strongly influence bacterial community structure, functional potential, and resistance gene expression. Arsenic was predominantly associated with amorphous iron minerals, resulting in high proportions of bioavailable fractions that significantly shaped microbial diversity and N, S, and P cycling functions (Liu, Yu et al.). In PAH-contaminated sediments, microbial community composition and sulfate-reducing bacteria were closely linked to contaminant concentrations and nutrient status, with direct implications for both natural attenuation capacity and human health risks (Lian et al.).

Iron and carbon cycling are intrinsically coupled across redox interfaces in both natural and anthropogenically impacted environments (Dong et al., 2023; Sheng et al., 2021, 2023a). The coupling between microbial metabolism and iron cycling was recently highlighted by column experiments simulating petroleum hydrocarbon attenuation in iron-rich aquifers (Zhang et al.). Rapid toluene degradation driven by iron-reducing consortia effectively constrained contaminant plume migration; however, this process concurrently generated mobile ferrous iron, leading to secondary groundwater contamination risks. The release of Fe may further alleviate microbial iron limitation, potentially reducing the energetic investment in siderophore production (Guo et al., 2024). These findings underscore the importance of considering biogeochemical by-products, rather than contaminant removal alone, in risk assessment and remediation strategies.

Beyond applied environmental contexts, this Research Topic also includes contributions that extend the conceptual boundaries of microbial-mineral interactions. A study exploring pyrite-catalyzed multiphase protocells demonstrated how mineral catalytic activity can induce hierarchical compartmentalization in coacervate-based droplets, offering new insights into mineral-enabled functional organization under prebiotic conditions (Ding et al.). This work conceptually reinforces the central role of mineral-organic interactions in shaping biochemical complexity across Earth's history.

At the organismal and genomic scale, comparative genomic analyses of *Ralstonia* spp. revealed distinct adaptive strategies enabling survival in aquatic environments, including loss of virulence-related systems and acquisition of specialized metabolic pathways (Liu, Mao et al.). These genomic traits reflect selective pressures typical of nutrient-limited and fluctuating aquatic habitats and provide a mechanistic link between environmental conditions and microbial functional evolution.

Collectively, microbial involvement in biogeochemical cycling and contaminant transformation at land-water ecotones is governed by a combination of nutrient availability, mineral associations, hydrological connectivity, and disturbance regimes. Importantly, microbial responses are often non-linear and mediated through functional redundancy, network interactions, and feedbacks with geochemical processes. These findings highlight the need to explicitly incorporate microbial dynamics into models of ecosystem functioning and environmental management.

Looking forward, future research should aim to integrate multi-omics approaches with high-resolution geochemical measurements, isotopic tracers, and hydrological modeling to better quantify microbial process rates and feedbacks across spatial and temporal scales. Beyond major elements, trace metals play critical yet often underappreciated roles in biogeochemical cycling in both ancient and modern environments (Dong et al., 2022; Sheng et al., 2023b; Zhou et al., 2024). Understanding microbial resilience and vulnerability will be essential for predicting ecosystem responses, guiding pollution mitigation, and sustaining biogeochemical services.
